# Pesticide Efficiency of Environment-Friendly Transition Metal-Doped Magnetite Nanoparticles

**DOI:** 10.3390/nano14020218

**Published:** 2024-01-19

**Authors:** Shamaila Shahzadi, Jalees Ul Hassan, Muhammad Oneeb, Saira Riaz, Rehana Sharif, Dayan Ban

**Affiliations:** 1 Waterloo Institute for Nanotechnology & Department of Electrical and Computer Engineering, University of Waterloo, Waterloo, ON N2L 3G1, Canada; 2 Physics Department, University of Engineering & Technology, Lahore 54890, Pakistan; 2018phdphy3@student.uet.edu.pk (J.U.H.); rehana@uet.edu.pk (R.S.); 3 Department of Parasitology, University of Veterinary & Animal Sciences, Lahore 54000, Pakistan; muhammad.oneeb@uvas.edu.pk; 4 Centre of Solid State Physics, Punjab University, Lahore 54590, Pakistan; saira.cssp@pu.edu.pk

**Keywords:** ferro fluids, pesticide, larvae, *Aedes aegypti*, magnetite, doping

## Abstract

This study explored the potential of Fe_3_O_4_, SnFe_2_O_4_, and CoFe_2_O_4_ nanoparticles as larvicidal and adulticidal agents against *Aedes aegypti* (*A. aegypti*) larvae and adults, which are vectors for various diseases. This research involved the synthesis of these nanoparticles using the coprecipitate method. The results indicate that CoFe_2_O_4_ nanoparticles are the most effective in both larvicidal and adulticidal activities, with complete mortality achieved after 96 h of exposure. SnFe_2_O_4_ nanoparticles also showed some larvicidal and adulticidal efficacy, although to a lesser extent than the CoFe_2_O_4_ nanoparticles. Fe_3_O_4_ nanoparticles exhibited minimal larvicidal and adulticidal effects at low concentrations but showed increased efficacy at higher concentrations. The study also revealed the superparamagnetic nature of these nanoparticles, making them potentially suitable for applications in aquatic environments, where *A. aegypti* larvae often thrive. Additionally, the nanoparticles induced observable damage to the gut structure of the mosquitoes and larvae, which could contribute to their mortality. Overall, this research suggests that CoFe_2_O_4_ nanoparticles, in particular, hold promise as environment-friendly and effective agents for controlling *A. aegypti* mosquitoes, which are responsible for the transmission of diseases such as dengue fever, Zika virus, and Chikungunya. Further studies and field trials are needed to validate their practical use in mosquito control programs.

## 1. Introduction

The rapid proliferation of deleterious parasites, pathogens, and arboviruses represents a pressing concern within the field of parasitology. Furthermore, the emergence of resistance in both disease parasites, as well as their arthropod vectors, has given rise to a significant challenge in the realm of tropical medicine. The expansion of maladies such as malaria, dengue fever, West Nile virus, Chikungunya, and arboviruses such as Japanese encephalitis and Zika virus, which are transmitted by vectors, demands stringent control measures employing environment-friendly methods. While numerous pesticides have been harnessed in the battle against these perilous vector-borne parasites, they have regrettably engendered severe environmental health repercussions. Consequently, the dearth of ecologically acceptable insecticides has evolved into a major cause for concern. Livestock, pets, wildlife, and humanity at large confront substantial risks posed by arthropod-borne diseases on a global scale [1]. The meticulous examination of mosquitoes as carriers of various parasites and pathogens has assumed paramount significance in the field of veterinary science. Each year, a staggering 390 million cases of dengue fever are reported, with a mere 96 million cases receiving effective clinical treatment [2]. Beyond dengue fever, warnings of numerous other mosquito-borne illnesses have been sounded. West Nile, Zika, and Chikungunya viruses experienced rapid proliferation in the Caribbean, Central America, and South America in 2013; these viruses fall within the same classification as dengue viruses [3].

*A. aegypti*, a diminutive black mosquito adorned with distinctive white leg stripes, predominantly inhabits tropical and subtropical climates across the globe, primarily between latitudes 35°S and 35°N. This species of mosquito thrives in regions where winter temperatures seldom dip below 10 °C, exhibiting a predilection for warm climates and rarely venturing beyond altitudes of 1000 m with lower temperatures. *A. aegypti* exhibits a strong association with human habitation and tends to concentrate its activities in close proximity to the locations where its eggs hatch [4]. Research findings have suggested that elevated temperatures may result in an augmented susceptibility to dengue outbreaks in temperate regions. Furthermore, analyses indicated a consistent increase of 1.5% in the global suitability for the renewal of the mosquito life cycle from 1950 to 2000, with projections indicating a potential acceleration to rates of 3.2% and 4.4% by 2050. Additionally, reductions in precipitation are observed to significantly influence the abundance of Aedes mosquitoes [5].

A discernible chasm exists between environmentally sustainable and recently introduced insecticidal agents. In response to this challenge, noteworthy strides have been made in the realm of nano-mosquitocides. Historically, conventional approaches to mosquito control have focused on reducing mosquito populations by employing diverse chemical agents and formulations based on Bacillus [6]. These methods aim to eliminate or mitigate mosquito breeding grounds. However, the escalated usage of chemical insecticides has wrought profound environmental and human health repercussions while also fostering the development of pesticide-resistant strains [7]. Although botanical pesticides do offer certain advantages, their availability as branded products in the market remains limited. Both the industry and researchers grapple with several impediments in their efforts to enhance the efficacy of these agents. Some promising plant-based substances exhibit pronounced toxicity toward arthropod vectors and pests. In recent years, the domain of nanoscience has catalyzed transformations across numerous sectors, including veterinary and medical sciences. Various nanoparticles (NPs), such as those composed of Ag [8,9], Au [10], TiO_2_ [11], Cu/CuO [12,13], and Zn [14], have undergone rigorous evaluation as potential pesticides against both mosquito larvae and adults, with their toxicological profiles meticulously scrutinized.

Magnetite demonstrates negligible environmental harm when used as an adulticidal or larvicidal agent. These NPs pose no detrimental effects on humans, animals, or plants during their application. Furthermore, their versatile application extends beyond pest control, encompassing roles in air purification, functioning as antimicrobial agents, serving as water treatment flocculants, and aiding in the segregation of carbon, heavy metals, selenium, radionuclides, and rare earth elements [15]. Notably, these NPs have demonstrated no adverse effects on critical factors such as germination rates, chlorophyll content, or plant growth. They do not elicit significant electrolyte release or cell death percentages in plants. Conversely, these nanoparticles exhibit a remarkable capacity to enhance enzyme activities and mitigate oxidative damage within plant systems [16]. Regrettably, no prior research endeavors have explored the toxicity of pure magnetite NPs and tin/cobalt-doped magnetite NPs toward arthropod vectors, such as mosquitoes. In the context of the present research, pure and tin/cobalt-doped magnetite NPs were synthesized according to the coprecipitate protocol and harnessed as novel pesticides against the *A. aegypti* vector.

## 2. Materials and Methods

### 2.1. Synthesis of Nanoparticles

The synthesis of Nano Ferro Fluids followed the coprecipitation methodology [17]. Three distinct samples of NPs were produced, namely, pure magnetite (Fe_3_O_4_) NPs, tin-doped magnetite (SnFe_2_O_4_) NPs, and cobalt-doped magnetite (CoFe_2_O_4_) NPs. The synthesis process employed iron chloride hexahydrate (FeCl_3_·6H_2_O), iron chloride tetrahydrate (FeCl_2_·4H_2_O), and sodium hydroxide (NaOH) as essential reagents. All the chemicals were purchased from Sigma Aldrich. The procedure commenced with the preparation of molar solutions for these raw materials. A 0.2 M FeCl_2_·4H_2_O solution was prepared by dissolving 2.535 g of FeCl_2_·4H_2_O in 100 mL of deionized water, while a 0.3 M FeCl_3_·6H_2_O solution was prepared by dissolving 4.87 g of FeCl_3_·6H_2_O in 100 mL of deionized water. Additionally, a 1.5 M NaOH solution was formed by dissolving 6 g of NaOH in 100 mL of water.

**Figure 1:**
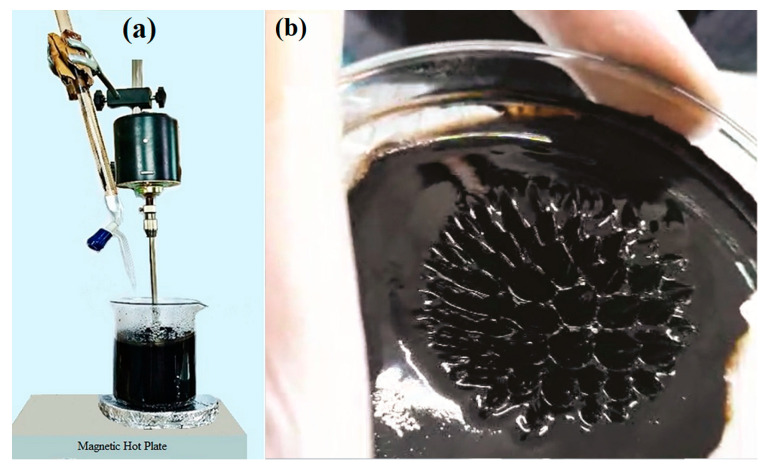
(**a**) Synthesis of Nanoparticles using the Coprecipitate Method. (**b**) Spikes formed by Nanoparticles under the Influence of the Magnetic Field.

Once the molar solutions were prepared, 30 mL each of the 0.3 M FeCl_3_·6H_2_O and 0.2 M FeCl_2_·4H_2_O solutions were combined in a 500 mL beaker. The resulting mixture was stirred for 10 min using an overhead stirrer and then heated on a hot plate, maintaining a temperature range of 60–70 °C, as illustrated in Figure 1a,b. Subsequently, the prepared 1.5 M NaOH solution was added drop by drop to the reaction mixture via a burette, with continuous stirring, for approximately 50 min until the solution’s pH reached approximately 12.

The synthesized NPs underwent a washing process in a petri dish with water, administered using a wash bottle. Magnetic separation was facilitated during washing, with the aid of a magnet, to isolate impurities from the NPs. To eliminate larger-sized particles, the NPs were passed through a 200 nm pore-size mixed cellulose ester membrane. Finally, the NPs were subjected to overnight drying in an oven at temperatures ranging from 80 to 90 °C, resulting in the acquisition of a finely powdered product. Equation (1) accurately describes the formation of Fe_3_O_4_ NPs.

(1) $${\mathrm{FeCl}}_{2}\cdot {4H}_{2}O+2{\mathrm{FeCl}}_{3}\cdot {6H}_{2}O+8\mathrm{NaOH}\to {\mathrm{Fe}}_{3}O_{4}↓+8\mathrm{NaCl}+14H_{2}O$$

The same protocol was followed for the synthesis of the SnFe_2_O_4_ and CoFe_2_O_4_ NPs. The chemical recipes of these NPs are written in Equations (2) and (3), respectively.

(2) $${\mathrm{xSnCl}}_{2}\cdot {2H}_{2}O+\left(1-x\right){\mathrm{FeCl}}_{2}\cdot {4H}_{2}{O+2\mathrm{FeCl}}_{3}\cdot {6H}_{2}O+8\mathrm{NaOH}\to {\mathrm{Sn}}_{x}{\mathrm{Fe}}_{3-x}O_{4}+8\mathrm{NaCl}+\left(20-2x\right)H_{2}O$$

(3) $${\mathrm{xCoCl}}_{2}\cdot {2H}_{2}O+\left(1-x\right){\mathrm{FeCl}}_{2}\cdot {4H}_{2}{O+2\mathrm{FeCl}}_{3}\cdot {6H}_{2}O+8\mathrm{NaOH}\to {\mathrm{Co}}_{x}{\mathrm{Fe}}_{3-x}O_{4}+8\mathrm{NaCl}+\left(20-2x\right)H_{2}O$$

### 2.2. Characterization Techniques

The Scanning Electron Microscopy (SEM) and Energy Dispersive X-rays (EDX) analyses of the Fe_3_O_4_, SnFe_2_O_4_, and CoFe_2_O_4_ NPs provided precise insights into their shape, size, and chemical composition. The elemental and morphological examination was conducted using a ZEISS Sigma 500 SEM. The EDX analysis revealed the presence of tin and cobalt elements incorporated into the magnetite structure. SEM images of all samples were captured at various magnifications. The crystalline, structural, and elemental analyses of the NPs were carried out using the High-Resolution X-ray Diffraction (HR XRD) analysis. The analysis was conducted with CoKα1/Kα2 radiation (λ = 1.7888/1.798 Å) in the 2θ range of 10° to 85° using the Bruker D2 Phaser. The magnetic properties of the NPs were characterized using a Vibrating Sample Magnetometer (VSM), which measures magnetic moment as a function of an applied magnetic field. The hysteresis graphs of the NPs proved instrumental in analyzing various magnetic properties, such as magnetic saturation, remanence, coercivity, or superparamagnetic behavior, with Lakeshore 7407 VSM used for the hysteresis plot measurements. The temperature-dependent magnetism of NPs was measured using the Superconducting Quantum Interference Device (SQUID Magnetometer, Quantum Design MPMS®3, San Diego, CA, USA). Additionally, Fourier Transform Infrared Spectroscopy (FTIR) was employed to determine the phases and chemical bonds within NPs. The FTIR spectra were recorded in the 400–1500 cm^−1^ range using Agilent Technology Cary 630 FTIR instrumentation.

### 2.3. Larvicidal Assay of Nanoparticles

The synthesized NPs were subjected to assessment for their larvicidal efficacy against *A. aegypti* larvae in accordance with the guidelines provided by the World Health Organization (WHO) [18]. To execute this, 5 g, 10 g, and 15 g of the NPs were dissolved individually in 100 mL of dechlorinated tap water, forming distinct working stocks. Subsequently, the concentrations of these solutions were adjusted to 50 mg/mL, 100 mg/mL, and 150 mg/mL, respectively. Different dilutions of these stocks were prepared in conjunction with deltamethrin (52918-63-5, Sigma Aldrich, St. Louis, MO, USA) employed as a positive control, while dechlorinated tap water served as the negative control. Each replicate consisted of 25 L3 stage larvae for all tested concentrations, and a total of 75 larvae was tested for each dose of NPs. Larval mortality rates were recorded at 24, 48, 72, and 96 h post-exposure, and the percentage mortality and standard deviation were calculated based on the average results from three replicates. The dissected midguts underwent fixation in a solution containing 4% paraformaldehyde (Sigma Aldrich) and phosphate-buffered saline (PBS) (BioPerformance Certified, Sigma Aldrich). Subsequently, these midgut specimens were scrutinized using a fluorescent microscope with a red filter that had an excitation and emission range of 495 nm and 517 nm, respectively, after being treated with the Phalloidin-iFluor 532 reagent (ab176755, Abcam, Shanghai, China).

### 2.4. Adulticidal Behavior of Nanoparticles

**Figure 2:**
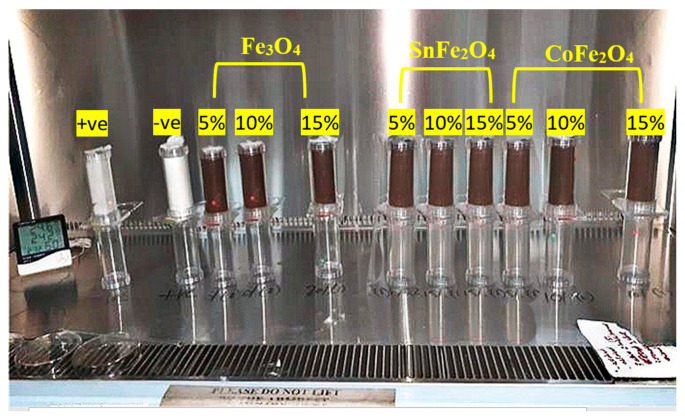
Experimental Setup to Check the Adulticidal Behavior of Pure and Doped Magnetite Nanoparticles against *A. aegypti* Mosquitos.

The optimal conditions for evaluating the adulticidal susceptibility of the Fe_3_O_4_, SnFe_2_O_4_, and CoFe_2_O_4_ NPs against *A. aegypti* entailed maintaining a temperature of 27 °C and a relative humidity exceeding 62%. The concentrations of NPs used in the adulticidal assay matched those employed in the larvicidal assessment. Adulticidal papers were immersed in these NP solutions, which contained 10% sucrose, and left to air dry at room temperature for several hours. Subsequently, these impregnated papers were securely affixed within test tubes, as illustrated in Figure 2. Three separate test tubes were employed for each NP concentration in the experimental setup. The dimensions of the paper coincided with those of the test tubes, ensuring a snug fit. Positive control tubes were treated with a lyophilized solution (at a concentration of 1 mL/100 mL water), whereas the negative control group received the same solvent as the insecticidal treatment. Each test tube was populated with precisely 25 adult mosquitoes, and a total of 75 adults were tested against each dose of NPs. Following the placement of the saturated paper, the test tube was sealed with either a screw-on or snap-on lid, designed to create an airtight seal that prevents mosquito escape and preserves the sample’s integrity. This meticulous sealing process is vital for precise mosquito population monitoring and surveillance. Subsequently, at intervals of 24 h, 48 h, 72 h, and 96 h, deceased mosquitoes were meticulously tallied. If the control group exhibited a mortality rate exceeding 20%, the entire experiment was deemed invalid. Deceased mosquitoes were carefully stored in appropriately labeled vials featuring small openings for moisture release. Before and after the exposure to NPs, mosquito guts dissections were performed, allowing us to observe the overall gut structure, through staining using the Phalloidin reagent, which specifically labels Actin filaments (F-actin) at the midgut of the mosquito. Adult *A. aegypti* of both genders can be encouraged to consume a diverse range of food options, such as fruit juices, milk, sugar solutions, sugar-containing chemicals, and various blood formulations [19]. The presence of sucrose and NPs within the dissected gut of *A. aegypti* was identified using an anthrone reagent [20] and HCl, respectively. Following the dissection of the midgut, a small quantity of HCl (Sigma Aldrich, 7647-01-0), typically a few microliters, was applied to the midgut of both larvae and adult *A. aegypti*. The solution’s color underwent a sudden transformation, turning brown, yellow, or green, due to the reaction illustrated below:

(4) $${\mathrm{Fe}}_{3}O_{4}+8\mathrm{HCL}\to {\mathrm{FeCl}}_{2}+{2\mathrm{FeCl}}_{3}+{4H}_{2}O$$

## 3. Results and Discussion

### 3.1. Surface Morphology and Elemental Analysis of Nanoparticles

**Figure 3:**
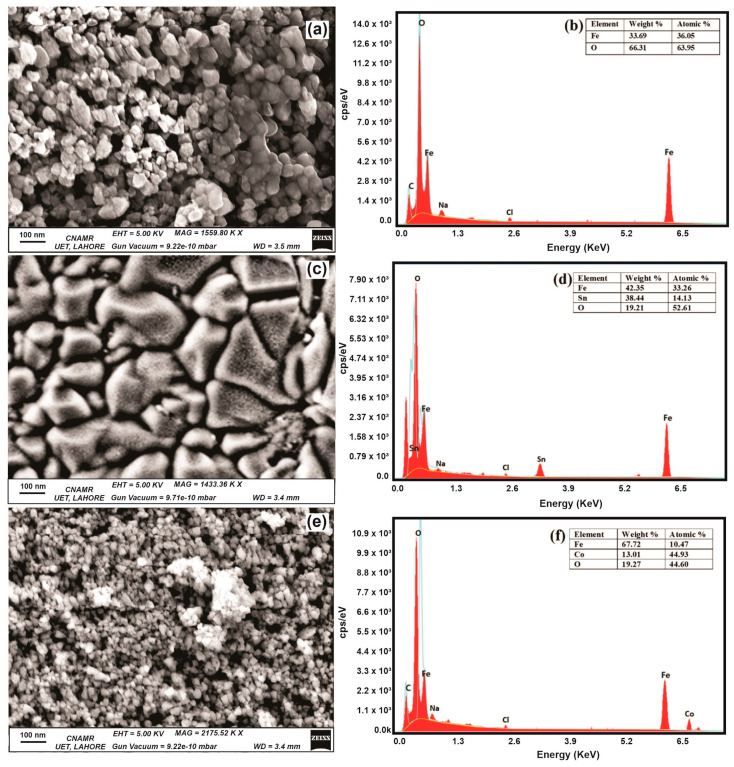
Scanning Electron Microscope Images of (**a**) Fe_3_O_4_ NPs, (**c**) SnFe_2_O_4_ NPs, and (**e**) CoFe_2_O_4_ NPs. EDX Spectrum of (**b**) Fe_3_O_4_ NPs, (**d**) SnFe_2_O_4_ NPs, and (**f**) CoFe_2_O_4_ NPs.

SEM images of NPs elucidate the morphology and dimensions of the NPs under scrutiny. Notably, the average dimensions for the Fe_3_O_4_, SnFe_3_O_4_, and CoFe_2_O_4_ NPs are recorded at 30 nm, 200 nm, and 20 nm, respectively. It is worth noting that Fe_3_O_4_ and CoFe_2_O_4_ NPs exhibit random morphology, whereas SnFe_2_O_4_ NPs display a more irregular, non-uniform shape, as shown in Figure 3a,c,e. This deviation in shape could be attributed to various factors in the synthesis process. The identification of Sn and Co ions relies on the analysis of EDX spectra, while the incorporation of these ions into the magnetite structure is verified through FTIR spectra. The EDX spectra of all samples are obtained in the range of 0–7.8 KeV, as depicted in Figure 3b,d,e. In the EDX of Fe_3_O_4_ NPs, peaks around 0.27 KeV, 0.7 KeV, 1.0 KeV, 2.6 KeV, and 6.4 KeV represent carbon, iron, sodium, chlorine, and iron, respectively. In the EDX of SnFe_2_O_4_ and CoFe_2_O_4,_ peaks around 3.5 KeV and 6.9 KeV specify doping of tin and cobalt ions within magnetite structure. Minor peaks observed for Na and Cl indicate the presence of NaCl salt in the product.

In the case of Fe_3_O_4_ NPs, the EDX analysis exclusively detects Fe and O atoms, indicating a high degree of phase purity. The relative proportions of these elements are elucidated in Figure 3b. For SnFe_2_O_4_ NPs, the EDX spectrum reveals a shift in atomic composition, with a decrease in the proportion of Fe atoms and a corresponding increase in Sn atoms.

### 3.2. Vibrating Sample Magnetometry of Nanoparticles

**Figure 4:**
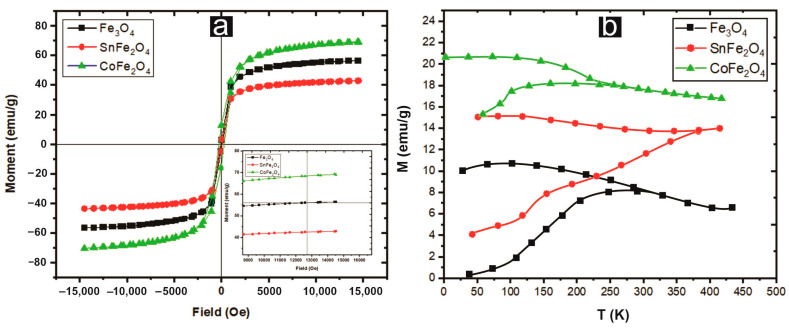
(**a**) Hysteresis curves of Nanoparticles (**b**) Magnetization-Temperature curves recorded in FC and ZFC modes for samples in the external magnetic field of 100 Oe.

The magnetic properties exhibited by these nanoparticles render them versatile in pesticide applications, as their capability to swiftly disengage from acoustic environments after larvicidal activity. The hysteresis curves of pure and doped magnetite NPs are measured at room temperature within a range of −15 kOe and +15 kOe. CoFe_2_O_4_ NPs showed a higher value of saturation magnetization (M_s_) (~68.98 emu/g) as compared to Fe_3_O_4_ and SnFe_2_O_4_ NPs, as displayed in Figure 4. It is clear from the results that these magnetic materials are enriched in M_s_ but deficient in remanence (M_r_) and coercivity (H_c_). The S-shape of the M-H curve, with negligible hysteresis and squareness ratio (M_r_/M_s_), suggests a superparamagnetic character, with a ratio range of 0.05 < M_r_/M_s_ < 0.5 characterizing a single-domain structure. The saturation magnetization has a direct dependence on the average particle size, as shown in Equation (5) [17].

(5) $$D=\left(6\delta +1\right)\frac{M_{B}}{M_{s}}$$

where ‘M_B_’ is the saturation magnetization of the bulk particles, ‘D’ is the average particle size, and ‘δ’ is the thickness of the paramagnetic shell. This equation indicates that NPs with small sizes possess high saturation magnetization and vice versa. The superparamagnetic behavior could also be confirmed by the nearly zero or minimum coercivity and remanence observed at room temperature, as shown in Table 1. The critical size that determines the superparamagnetic behavior of NPs is described in Equation (6) [21].

(6) $$D_{s}=\frac{k_{B}T}{K}=28.65\;\mathrm{nm},$$

where D_s_ is the critical diameter of the particles, k_B_ is Boltzmann’s constant, ‘K’ is the anisotropy constant (for Fe_3_O_4_ NPs, it is $0.44\times 10^{4}$ J/m^3^), and T is the absolute temperature. It is observed that Fe_3_O_4_ and CoFe_2_O_4_ NPs have small particle sizes and have superparamagnetism. Furthermore, doping of non-magnetic tin decreases the saturation magnetization and leads to larger particle sizes.

Figure 4b depicts the Zero field cooling magnetization (M_ZFC_) and Field cooling magnetization (M_FC_) curves of three samples acquired at a magnetic field strength of 100 Oe. Notably, at a temperature of 300 K (blocking temperature), a clear separation between the ZFC and FC curves of Fe_3_O_4_ NPs becomes apparent. At this blocking temperature, a transition manifests itself, shifting from a ferromagnetic (FM) state for the temperature below T_B_ to a superparamagnetic (SPM) state for temperatures exceeding T_B_. Consequently, the magnetic behavior of Fe_3_O_4_ and CoFe_2_O_4_ NPs at 300 K is predominantly SPM, whereas SnFe_2_O_4_ NPs primarily exhibit FM properties. Detailed values for T_B_ for all the samples are provided in Table 1.

### 3.3. X-ray Diffraction Analysis of Nanoparticles

**Figure 5:**
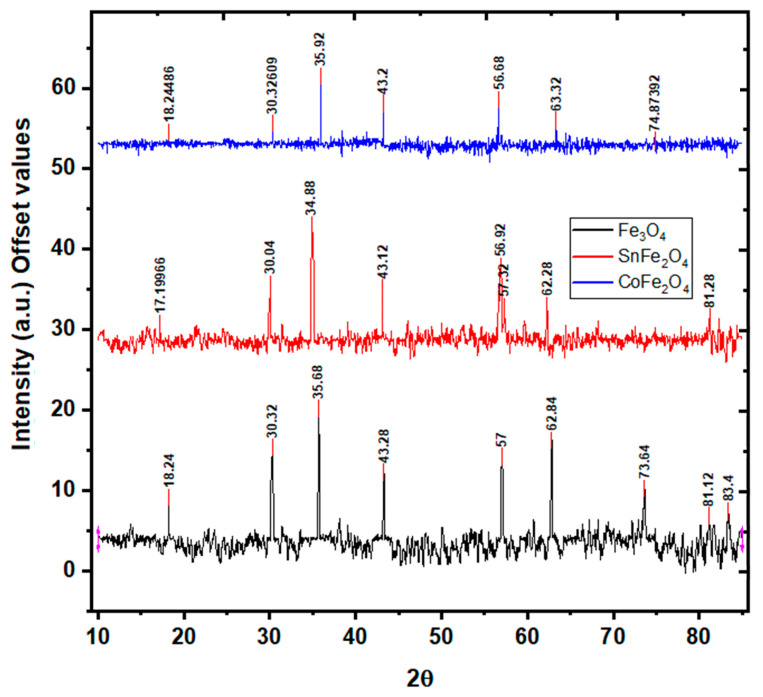
X-ray Diffraction Analysis of Fe_3_O_4_ NPs, SnFe_3_O_4_ NPs, and CoFe_2_O_4_ NPs.

During the synthesis process, different phases of iron oxide NPs, such as hematite and maghemite, could be formed. The verification of the magnetite phase synthesis is substantiated using XRD analysis. XRD analysis depicting the characteristics of Fe_3_O_4_, SnFe_2_O_4_, and CoFe_2_O_4_ is illustrated in Figure 5. The discerned critical peaks for Fe_3_O_4_ NPs, located at 30.32°, 35.68°, 43.28°, 57°, and 62.84°, correspond to the (220), (311), (400), (511), and (440) crystallographic planes, respectively. These peaks unequivocally signify the cubic inverse spinel structure of Fe_3_O_4_ NPs, a confirmation derived from the comparison with the JCPDS 19-0629 standard pattern [22]. Notably, the XRD spectrum of Fe_3_O_4_ NPs reveals an average crystallite size of 8 nm, computed using the Scherer formula. Turning to the XRD patterns of SnFe_2_O_4_ NPs, the detected peaks at 30°, 34.8°, 43.1°, 56.9° and 62.28° can be assigned to the (220), (311), (400), (511), and (440) planes, respectively. These peaks affirm the face-centered cubic (FCC) structure and Fd-3m space group, an authentication corroborated by reference to the ICSD standard pattern (ICSD No. 98-015-8742) [23]. The exclusive presence of these significant peaks is consistent with the phase purity of the NPs. The XRD analysis of SnFe_2_O_3_ NPs yields an average crystallite size of 12 nm. Interestingly, in the case of SnFe_2_O_4_ NPs, a subtle peak shift towards smaller diffraction angles is observed, attributable to the relatively smaller ionic size of Fe^+2^ (1.18 Å) as compared to Sn^+2^ ions (0.77 Å) [23]. Lastly, the XRD peaks detected for CoFe_2_O_4_ NPs, positioned at 30.32°, 35.92°, 43.2°, 56.68°, 63.32°, and 74.87°, correspond unambiguously to the (220), (311), (400), (511), and (440) planes, as delineated in Figure 5. These findings collectively validate the structural characteristics of CoFe_2_O_4_ NPs. Notably, the Scherer formula computes the crystallite size of CoFe_2_O_4_ NPs to be 6 nm.

### 3.4. Fourier Transform Infrared Spectroscopy of Nanoparticles

**Figure 6:**
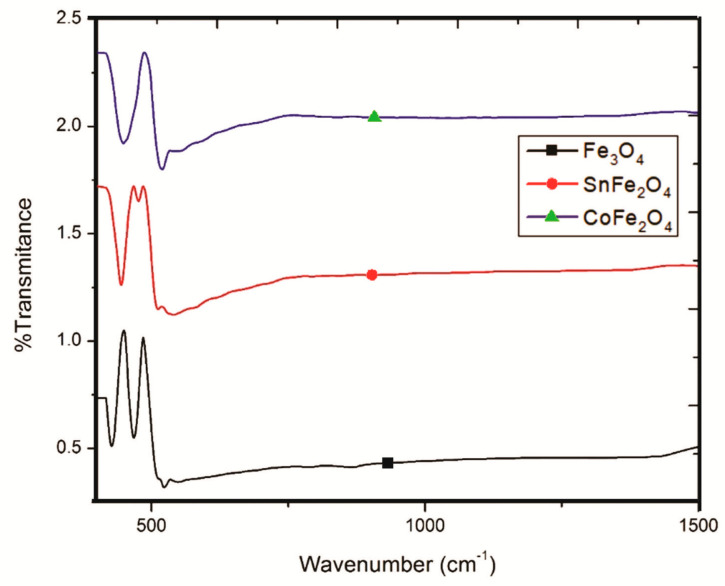
FTIR Analysis of Fe_3_O_4_ NPs, SnFe_2_O_4_ NPs, and CoFe_2_O_4_ NPs.

FTIR spectra play a pivotal role in facilitating the comprehensive analysis of functional groups present in NPs, as visually depicted in Figure 6. These FTIR spectra have been meticulously scrutinized within the wavenumber range of 250 to 1500 cm^−1^. Remarkably, the spectra of the NPs exhibit remarkable purity, devoid of any extraneous peaks. In the case of Fe_3_O_4_ NPs, the discernible presence of two distinct peaks at approximately 426.8 cm^−1^ and 466.5 cm^−1^ is indicative of stretching vibration modes associated with the metal oxide absorption band (pertaining to Fe^+3^-O and Fe^+2^-O bonds within the crystallite lattice of Fe_3_O_4_ NPs). Conversely, the SnFe_2_O_4_ spectrum offers compelling evidence of Sn^+2^ doping within the magnetite structure, underscored by the appearance of a distinctive peak at approximately 537.4 cm^−1^, signifying the presence of O-Sn-O bonds.

Additional noteworthy peaks at approximately 443.8 cm^−1^ and 475 cm^−1^ are attributed to Sn^+2^-O and Fe^+3^-O bonds within the inverse spinel structure of these NPs. Turning our attention to the FTIR spectrum of CoFe_2_O_4_ NPs, it reveals a conspicuous broad peak at 448.1 cm^−1^, which is attributed to Fe^+3^/Co^+2^-O bonds, while a discernible peak at 553 cm^−1^ is indicative of O-Co^+2^-O bonds.

### 3.5. Larvicidal Efficiency of Nanoparticles

The mortality percentages resulting from the exposure of *A. aegypti* larvae to varying concentrations of NPs are determined using Equation (7) [18].

(7) $$\mathrm{Percentage}\;\mathrm{Mortality}=\frac{\mathrm{Mortal}\;\mathrm{Larvae}}{25}\times 100\;$$

**Figure 7:**
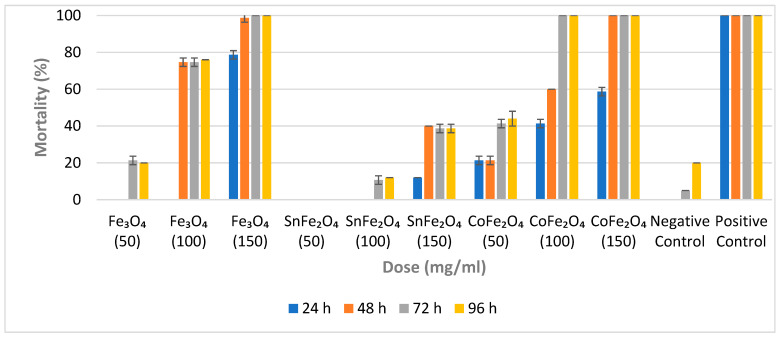
Percentage Mortality of Fe_3_O_4_ NPs, SnFe_2_O_4_ NPs, and CoFe_2_O_4_ NPs against *A. aegypti* Larvae After 24, 48, 72 and 96 h.

For each concentration of a sample, a set of triplicate cups is employed, and the average outcome, along with the standard deviation (SD), is calculated. The impacts of these NPs on *A. aegypti* larvae are evaluated at intervals of 24 h, 48 h, 72 h, and 96 h, respectively. Fe_3_O_4_ NPs exhibit no larvicidal effects at a concentration of 50 mg/mL during the initial 24 and 48 h. However, a slight larvicidal effect is observed at this low concentration after 72 and 96 h. This suggests that despite their minute particle size, a concentration of 50 mg/mL is inadequate to yield significant larvicidal efficacy, as shown in Figure 7. Notably, a Fe_3_O_4_ NP concentration of 100 mg/mL yields a mortality rate of 76% after 96 h. The highest larval mortality rate (100%) for Fe_3_O_4_ NPs is achieved at a concentration of 150 mg/mL within 72 h.

In contrast, SnFe_2_O_4_ NPs display no activity at a concentration of 50 mg/mL, possibly due to both their low concentration and larger particle size. Even at a concentration of 100 mg/mL, SnFe_2_O_4_ NPs do not induce any larval mortality within the initial 48 h. However, a 10.67% mortality rate is observed after 72 h at this concentration. The highest mortality rate of 40% is observed for SnFe_2_O_4_ NPs at a concentration of 150 mg/mL after 48 h.

The minimal concentration of CoFe_2_O_4_ NPs does not result in a 100% mortality rate; however, concentrations of 100 mg/mL and 150 mg/mL of CoFe_2_O_4_ NPs exhibit complete mortality within 72 h and 48 h, respectively. Since all NPs are synthesized using identical protocols, CoFe_2_O_4_ NPs display characteristics such as small particle size, substantial magnetization, and potent larvicidal properties. Cobalt-doped magnetite NPs appear to be promising candidates for employment in aquatic environments, particularly those with a high prevalence of *A. aegypti* eggs or larvae. Furthermore, CoFe_2_O_4_ NPs can be easily separated from aquatic environments through the use of a powerful magnet.

### 3.6. Adulticidal Efficacy of Nanoparticles

The percentage mortalities of various concentrations of NPs against *A. aegypti* adults are calculated by Equation (8) [24].

(8) $$\mathrm{Percentage}\;\mathrm{Mortality}=\frac{\mathrm{Mortal}\;\mathrm{Adults}}{25}\times 100$$

**Figure 8:**
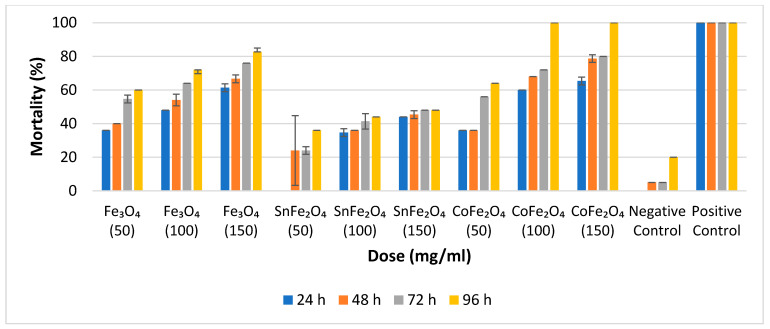
Percentage Mortality of Fe_3_O_4_ NPs, SnFe_2_O_4_ NPs, and CoFe_2_O_4_ NPs Against *A. aegypti* Mosquitos after 24, 48, 72, and 96 h.

For the assessment of adulticidal efficacy at various concentrations, triplicate tubes are employed. The average outcomes pertaining to adulticidal efficiency are determined at 24 h, 48 h, 72 h, and 96 h, respectively, as illustrated in Figure 8.

Notably, there is a direct correlation between nanoparticle concentration and adulticidal efficacy; an escalation in nanoparticle concentration is accompanied by an increase in mortality rates. Specifically, at a concentration of 50 mg/mL, Fe_3_O_4_ and CoFe_2_O_4_ NPs exhibit approximately equivalent results, while at 100 mg/mL, Fe_3_O_4_, SnFe_2_O_4_, and CoFe_2_O_4_ NPs demonstrate mortality rates of 72%, 48%, and 100%, respectively. Remarkably, the achievement of 100% mortality, signifying the demise of all 25 adult mosquitoes, is exclusively attained with CoFe_2_O_4_ NPs at concentrations of 100 mg/mL and 150 mg/mL, following a 96 h duration. The mortality data for all 25 adult *A. aegypti* mosquitoes is presented in Figure 8.

### 3.7. Damages to the Gut Induced by Nanoparticles

**Figure 9:**
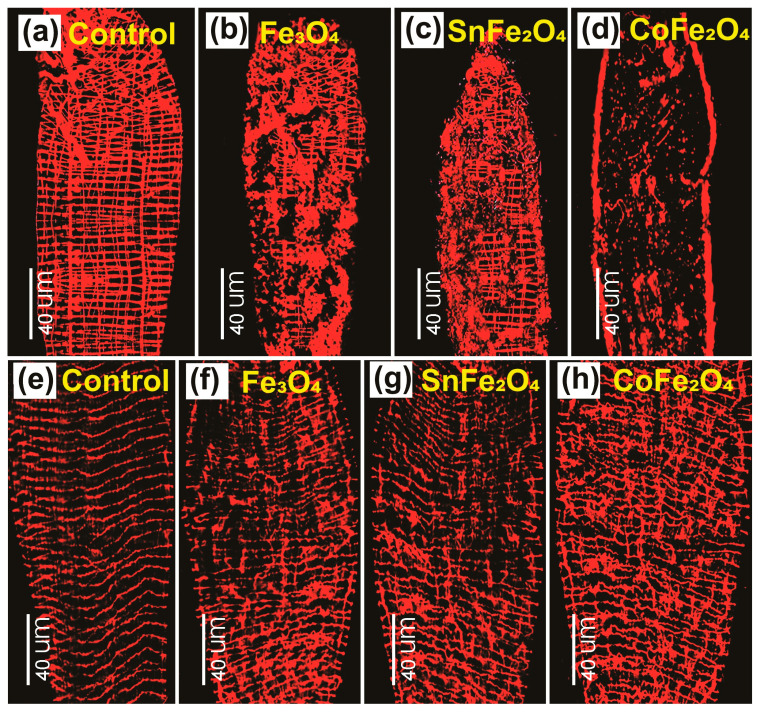
Phalloidin Staining Micrograph of Dissected Guts of *A. aegypti* taken using the Leica Fluorescent Microscope: (**a**) Mid-gut of Control Larva, (**b**) Larva Fed on Fe_3_O_4_ NPs, (**c**) Larva Fed on SnFe_2_O_4_ NPs, (**d**) Larva Fed on CoFe_2_O_4_ NPs, (**e**) Midgut of Control Adult, (**f**) Mosquito Fed on Fe_3_O_4_ NPs, (**g**) Mosquito Fed on SnFe_2_O_4_ NPs, and (**h**) Mosquito Fed on CoFe_2_O_4_ NPs.

The established gut model of *A. aegypti* larvae and adults provides a platform for studying the toxicity resulting from exposure to NPs [25]. Figure 9a notably exhibits a grid-like morphology composed of bands consisting of single- or multiple-muscle fibers. It is important to note that the orientation of these bands is not consistent across the larval midgut. The impact caused by the Fe_3_O_4_, SnFe_2_O_4_, and CoFe_2_O_4_ NPs is depicted in Figure 9b–d. It is observable that these nanoparticles possess the ability to inflict damage on larval guts, thus presenting a valuable model for analyzing the effects of such damage on the structure and physiology of the gut.

Actin filaments, integral components of the cytoskeleton, are microfilaments that play essential roles in both muscle and non-muscle cells as part of the contractile apparatus. Within cellular contexts, F-actin assumes multifaceted functions, encompassing structural support, mechanical contributions, and even enzymatic involvement [26]. Figure 9e illustrates the sequenced arrangement between the two strands of F-actin within the gut of control mosquitoes. This particular sequence represents a stabilized configuration of F-actin filaments comprising intrastrand and interstrand components. Conversely, in the guts of mosquitoes subjected to a diet enriched with Fe_3_O_4_, SnFe_2_O_4_, and CoFe_2_O_4_ NPs in conjunction with sucrose, noticeable distortions are observed. Furthermore, the F-actin filaments in these experimental cases display a diminished degree of uniformity when juxtaposed with those in the control mosquito gut. Among these treatments, CoFe_2_O_4_ NPs exhibit the most pronounced morphological distortions.

The impact of NPs on the gut reveals a notable variance between the larval and adult stages, indicating a more significant impairment in the larval gut. This discrepancy is attributed to the size and quantity of muscle bands within the larval gut. Notably, larval muscles have a smaller size, and their higher abundance contributes to a more vulnerable gut structure. Specifically, the spacing between longitudinal muscle bands is considerably wider in 3rd instar larvae (measuring at 30.5 ± 0.6 µm) compared with 4th instar larvae (25.4 ± 1.3 µm) or adults [27]. This increased spacing creates a more accessible pathway for NPs to invade the larval gut compared with the more densely packed arrangement in older stages and adult mosquitoes.

### 3.8. Comparative Study

Numerous investigations have been undertaken within the realm of entomology exploring the efficacy of various nanoparticles, such as gold, silver, copper, and titanium, in combating both the adult and larval stages of *A. aegypti.* These NPs lack magnetic properties and cannot be isolated from their aquatic environment. Although these NPs do not have any side effects for humans, animals, and plants, long-term exposure to these NPs could be harmful [28]. A big problem has been associated with the chemical and green synthesis process of these NPs. All chemical synthesis processes contain some undesired ingredients in the product; for example, the chemical synthesis of silver NPs is governed by a simple equation:

$${2\mathrm{AgNO}}_{3}+{2\mathrm{NaBH}}_{4}\to {2\mathrm{Ag}+B}_{2}H_{6}+H_{2}↑{\mathrm{NaNO}}_{3}$$

The presence of diborane and sodium nitrate in the product results in the impurity of silver NPs. For magnetic nanoparticles, purity has been attained through the separation of particles using a powerful magnet. The toxicity impact of green-synthesized Ag [8,9], Au [10], TiO_2_ [11], Cu/CuO [12,13], and ZnO [14] NPs on *A. aegypti* adults and larvae has been investigated by many research groups in 13 countries, mainly in India, Italy, and Saudi Arabia [29]. A major problem concerning green synthesis is the purity of NPs. The synthesis of these NPs is followed by plant extract (fruit, seed, stem, leaf, flower, peel, root, rhizome, and bark), which contains a lot of other constituents; for example, apple extract has hydroxy acids, phenol groups, and vitamin C [30]. Every chemical and green synthesis of NPs is accompanied by a lot of unwanted ingredients, which create doubts about the achieved results. Therefore, the ultrapurity of magnetic NPs makes them unique among all others.

## 4. Conclusions

The coprecipitate approach is a generic one for the synthesis of nanoparticles. It is favorable for synthesizing CoFe_2_O_4_ NPs using the coprecipitate method because this method provides small particle sizes and strong magnetic behavior to NPs. The EDX and FTIR analysis characterizes tin/cobalt doping, which reveals that Sn^+2^/Co^+2^ ions replace Fe^+2^ at A and B sites. Pure and doped magnetite NPs have FCC inverse spinel structures and are members of the Fd-3m space group. These NPs are superparamagnetic, with higher values of saturation magnetization. These nanoparticles demonstrate adulticidal and larvicidal effects against *A. aegypti* mosquitoes. Notably, among these nanoparticles, cobalt-doped magnetite nanoparticles exhibit significant adulticidal and larvicidal behavior. It is evident from the results that *A. aegypti* is more vulnerable in the larvae stage and can be killed easily compared with the adults. It is observed that *A. aegypti* larvae and adults are killed by the distortion of gut muscles.

## Figures and Tables

**Table 1:** Saturation Magnetization (M_s_), Remanence (M_r_), Coercivity (H_c_), and Blocking Temperature of Nanoparticles.

Sample (NPs)	M_s_ (emu/g)	M_r_ (emu/g)	M_r_/M_s_	+H_c_ (Oe)	−H_c_ (Oe)	$\frac{+H_{c}+-H_{c}}{2}$	T_B_ (K)
Fe_3_O_4_	56.42	4.5	0.080	1.03	1.99	1.51	300
SnFe_2_O_4_	42.00	1.7	0.040	71.85	118.44	105.145	387
CoFe_2_O_4_	68.98	13.2	0.191	0.37	0.89	0.5375	262

## Data Availability

The data that support the findings of this study are available on request from the corresponding author (S.S.).
